# Metastatic Follicular Carcinoma of the Thyroid: A Case Report

**DOI:** 10.7759/cureus.65609

**Published:** 2024-07-28

**Authors:** Vasundara Gopalan, Darshana Tote, Swati G Deshpande, Abhilasha Bhargava, Amol A Gupta

**Affiliations:** 1 General Surgery, Jawaharlal Nehru Medical College, Datta Meghe Institute of Higher Education and Research, Wardha, IND

**Keywords:** iodine deficiency, follicular carcinoma, neck swelling, neck lump, papillary thyroid carcinoma

## Abstract

Neck lumps may indicate metabolic disorders of the parathyroid and thyroid glands commonly present in the anterior aspect of the neck. Some neck lumps are detected as follicular thyroid cancer. Follicular thyroid cancer is a malignant epithelial tumor that shows evidence of follicular cell differentiation but lacks the characteristic nuclear features of papillary thyroid carcinoma (PTC). Iodine-deficient regions have higher rates of follicular carcinoma. There has been a decline in the incidence of this kind of tumor in recent years. As with PTC, prior radiation therapy increases the risk of follicular cancer, but to a lesser extent. In some cases, patients have distant metastases that involve the bones. Follicular cancer is divided into minimally invasive and widely invasive types. Early detection is important. Treatment usually consists of thyroidectomy and radioactive iodine therapy, and hormone replacement therapy may be necessary. Fine needle aspiration cytology is an efficient and cost-effective tool for the diagnosis of neck swelling and has the potential to diagnose the mass. We report the case of a 60-year-old Indian woman who had been experiencing neck swelling for the last 12 years. The neglected neck mass was confirmed as a hyperechoic mass with microcalcifications on ultrasound, representative of metastatic follicular thyroid carcinoma, which further spread to the ribs and the vertebrae. The patient was followed without complications.

## Introduction

The function of the thyroid gland is to produce thyroid hormones that the body needs to perform various metabolic functions. Follicles make up the thyroid gland and are the functional and structural units of the thyroid gland. These cells can grow abnormally and lead to serious follicular disease. One of the most prevalent endocrine tumors, thyroid cancer can be classified as differentiated or undifferentiated [1]. Atypical cancers include those that are papillary and follicular, and typical types include those that are medullary and anaplastic. Follicular thyroid cancer is the second most common type and accounts for 10-15% of all thyroid cancers [2]. Up to half of follicular cancer cases show *RAS* mutations, one-third show *PAX-PPARG* mutations, and only 3% show both [2,3]. Ultrasound imaging (USG) is the primary modality of choice, which shows hypoechoic soft tissue nodules with ill-defined borders, microcalcifications, and a tubular pattern with abundant internal flow, unlike the surrounding nodules. Computed tomography (CT) and USG-guided fine-needle aspiration (FNA) or core biopsy can be utilized to diagnose primary thyroid cancer quickly [3]. Early diagnosis reduces delay in treatment and prognosis gets better.

## Case presentation

**Figure 1:**
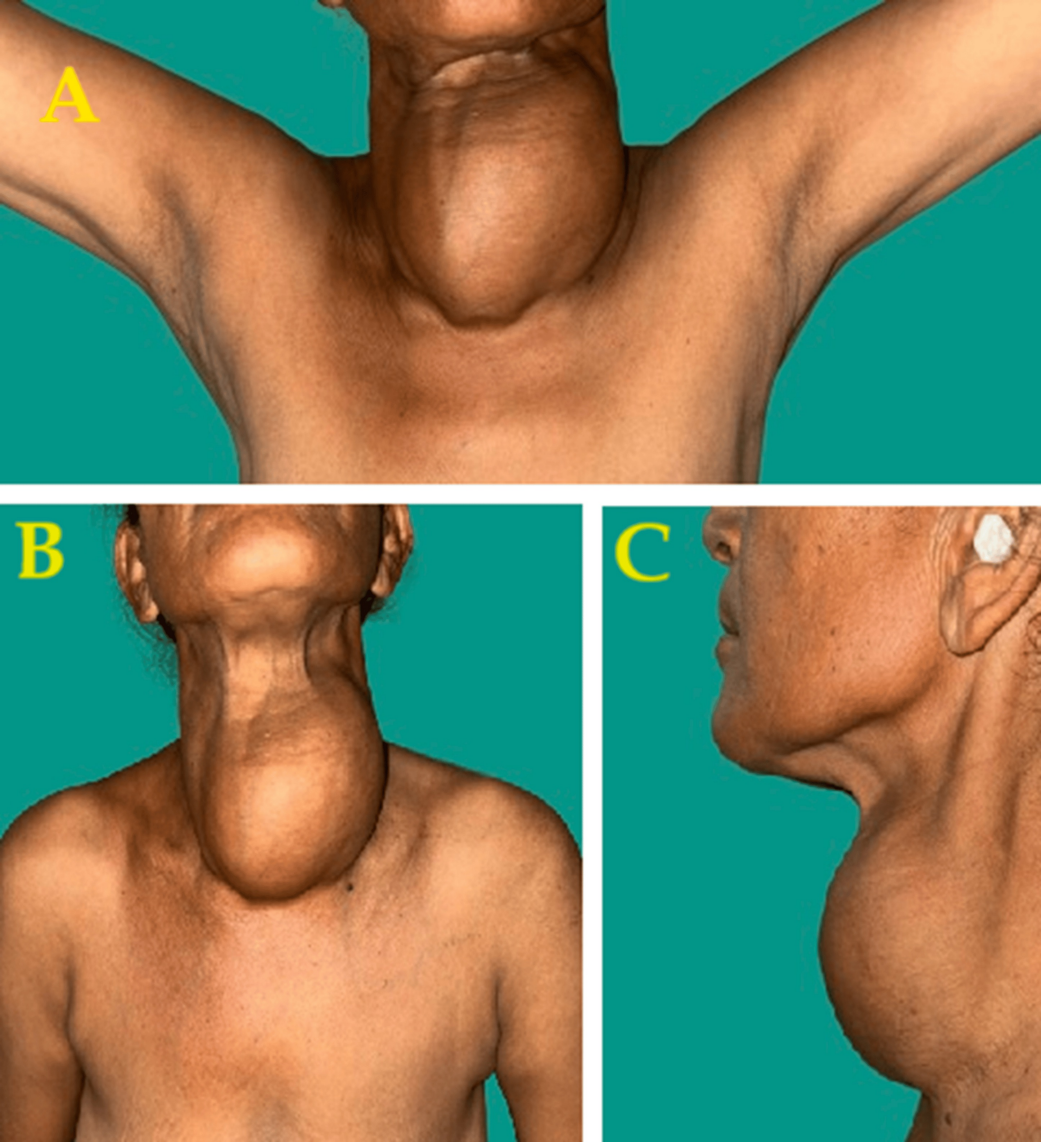
Physical presentation of the swelling of the neck

A 60-year-old Indian female presented with swelling across her anterior neck region for 12 years, which was insidious onset initially pea-sized. Painless progression of the swelling was noticed in the last two years. The patient also complained of chest wall pain and swelling. It was not associated with dyspnea, hand tremors, breathlessness, voice change, palpitation, or any co-morbidities. Clinically, the swelling was solitary and firm in consistency on the anterior aspect of the neck, measuring 12 x 8 cm in size (Figure 1).

**Figure 2:**
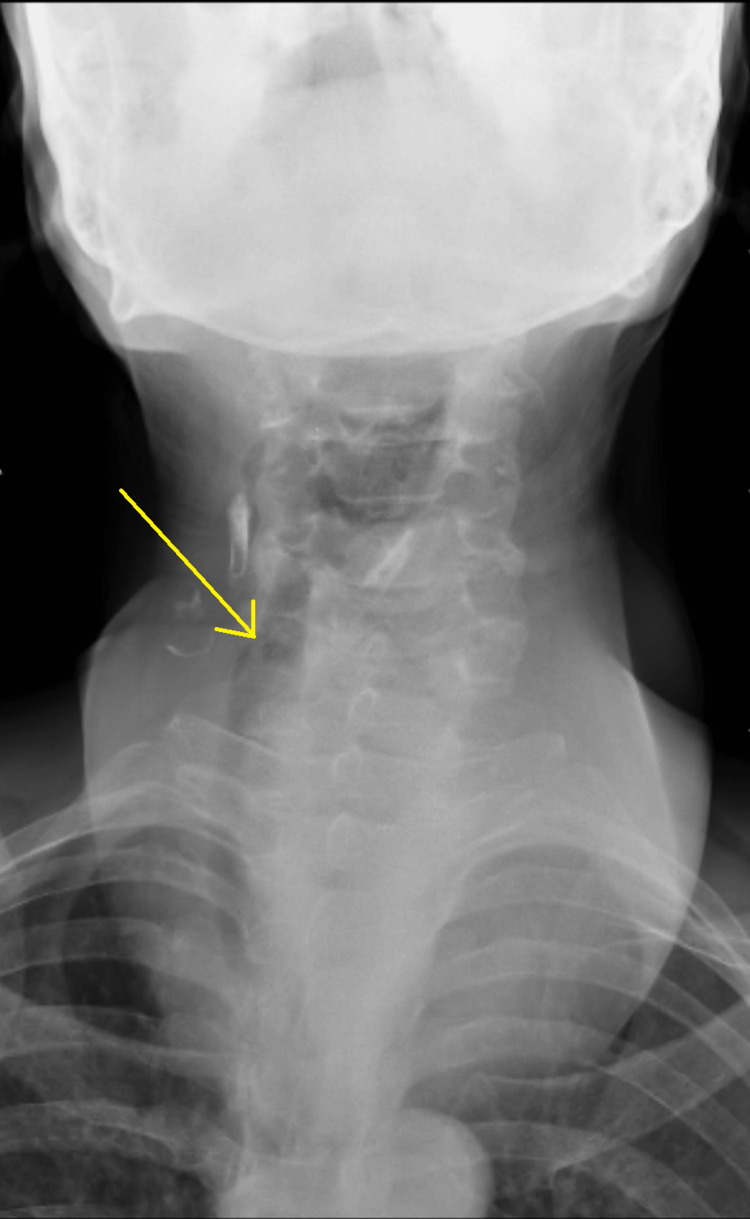
X-ray neck anteroposterior view showing the tracheal deviation

The swelling involved both lobes of the thyroid gland and the isthmus, along with evidence of engorged veins. Retrosternal extension of the gland noted. The patient had no palpable axillary lymph nodes. The patient's thyroid profile was suggestive of euthyroidism with thyroid stimulating hormone (TSH) (0.746 μIU/ml), FT3 (2.77 pg/ml), and FT4 (0.78 ng/dl) values. The anterior view of the neck revealed a well-defined swelling over the anterior aspect of the neck, more toward the right. There was evidence of a mass effect in the form of slight tracheal deviation. On the anteroposterior view, it was suggestive of tracheal deviation toward the right side (Figure 2).

**Figure 3:**
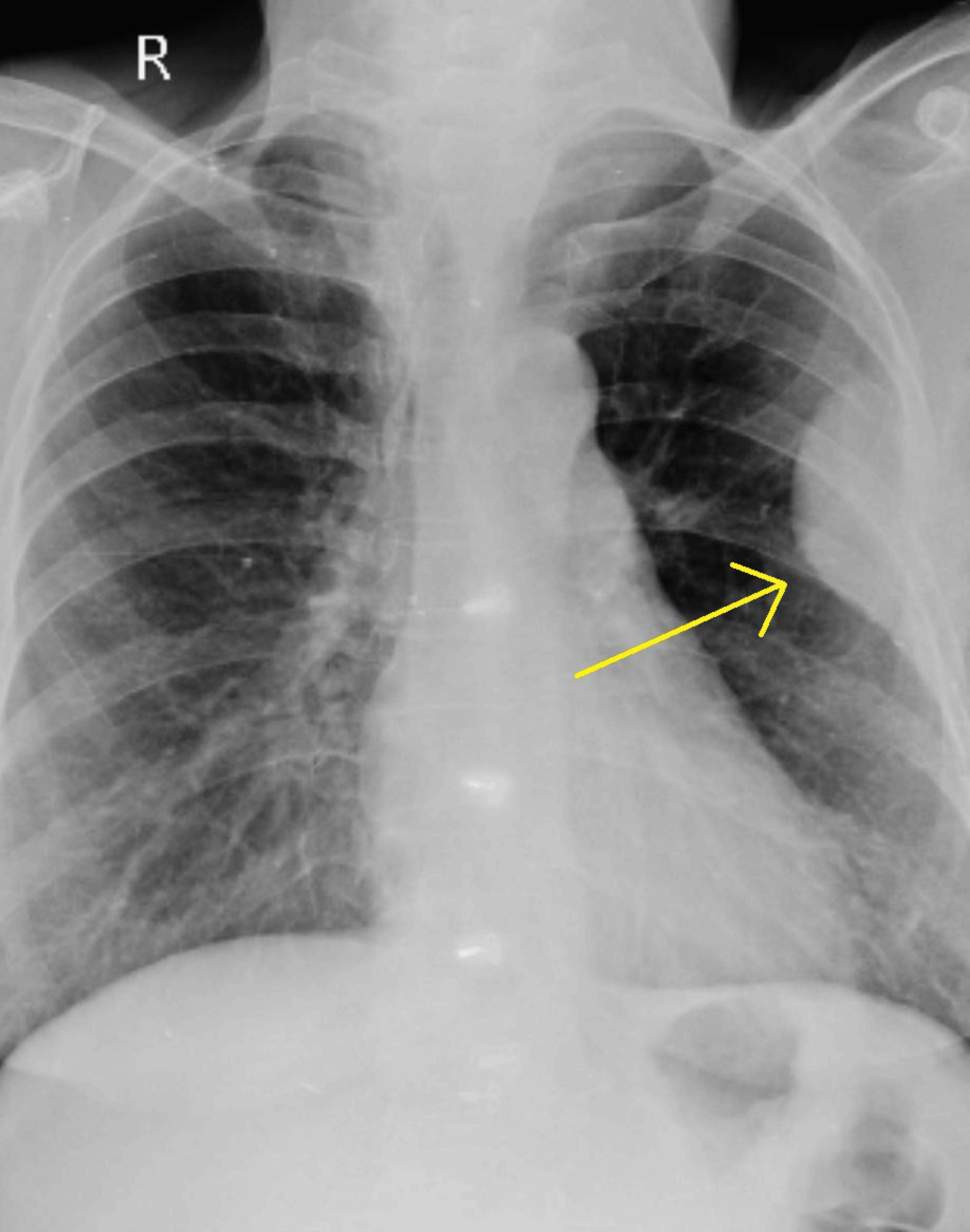
Posteroanterior view X-ray with a possibility of a pleural-based mass

Chest radiograph revealed left-sided pleural-based mass extending along the fifth, sixth, and seventh ribs (Figure 3).

**Figure 4:**
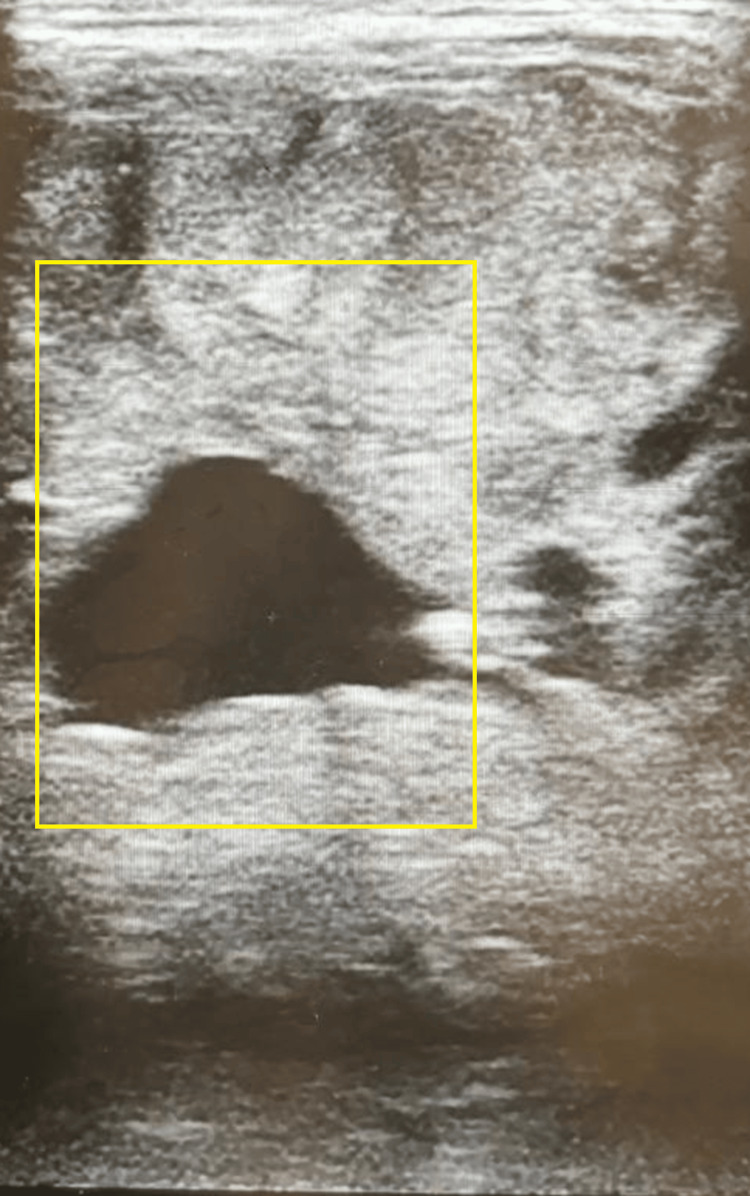
Ultrasonography of the neck showing anechoic cystic lesion with multiple separations

USG of the neck revealed an anechoic cystic lesion with multiple septations (Figure 4) within and an isoechoic lesion in the right lobe. A well-defined isoechoic lesion with peripheral calcification was also seen in the right lobe. Isthmus appeared bulky with a heterogenous echo texture. The left lobe of the thyroid appeared bulky, heterogeneous in texture, and multiloculated causing mass effect.

**Figure 5:**
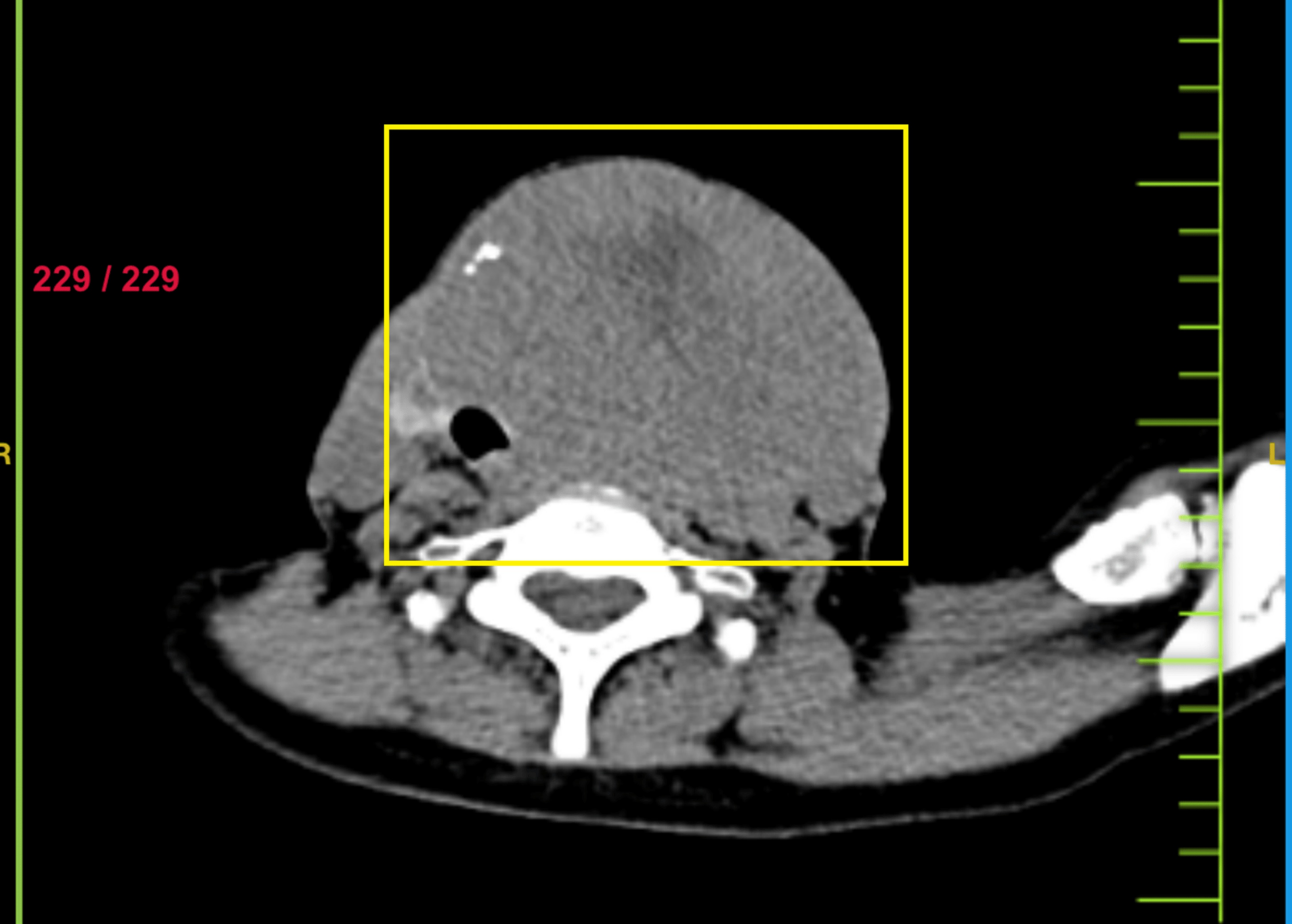
Non-contrast computed tomography image showing thyroid mass with tracheal deviation to the right

**Figure 6:**
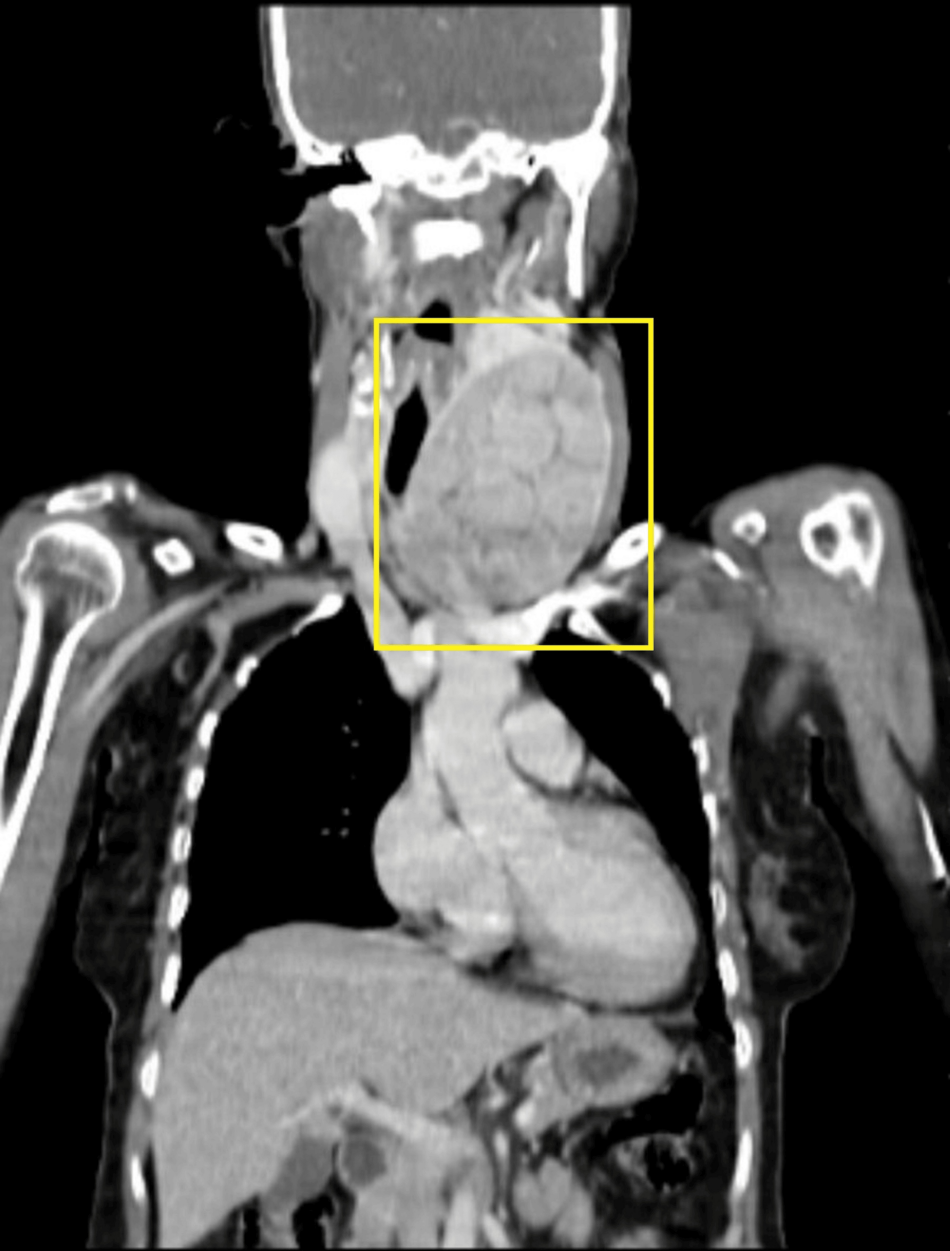
CECT showing the thyroid nodule (coronal view) showing an oval-shaped, well-circumscribed, multinodular soft tissue lesion that shows vivid enhancement CECT: contrast-enhanced computed tomography

**Figure 7:**
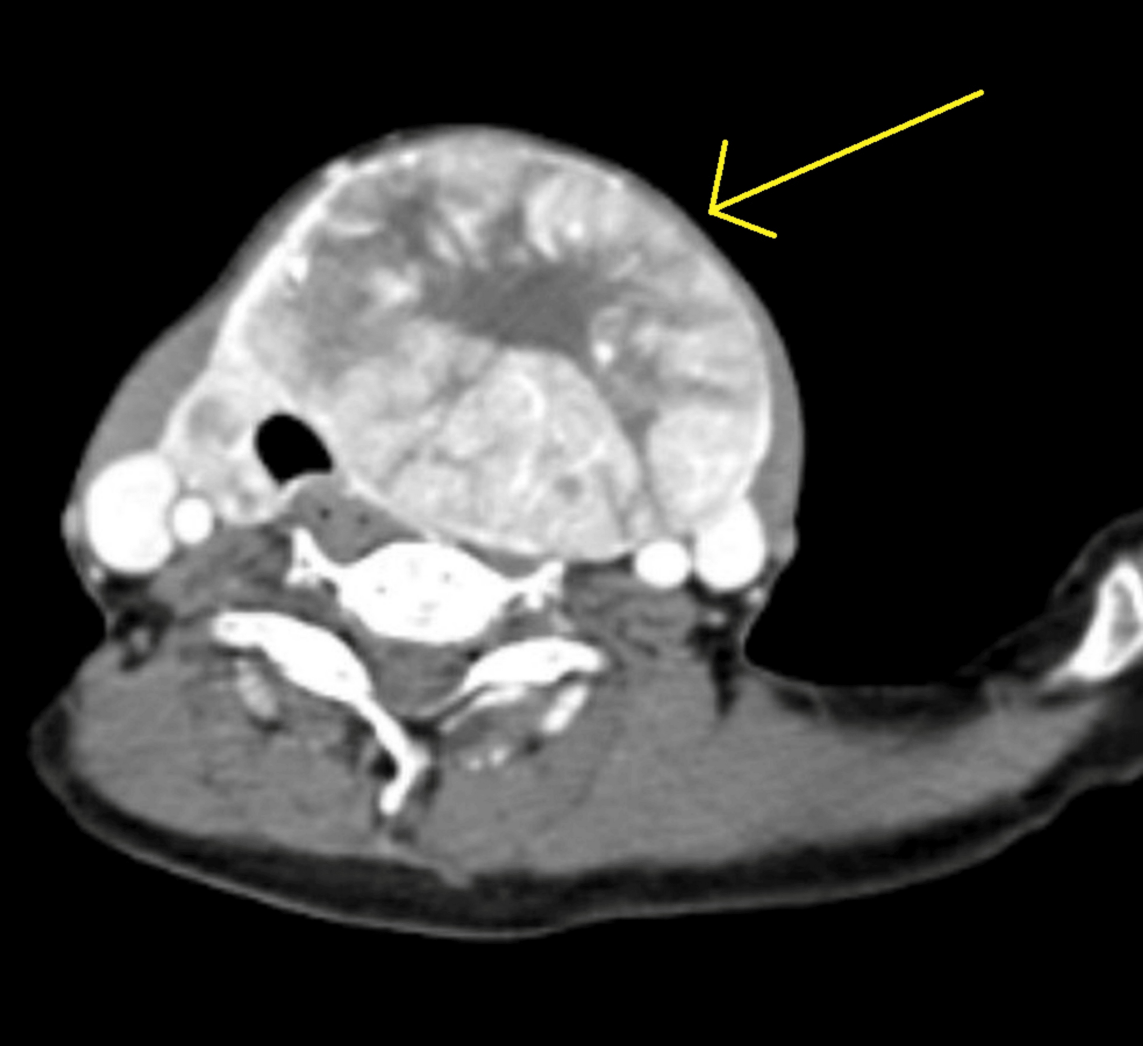
CECT showing the thyroid nodule (axial view) CECT: contrast-enhanced computed tomography

**Figure 8:**
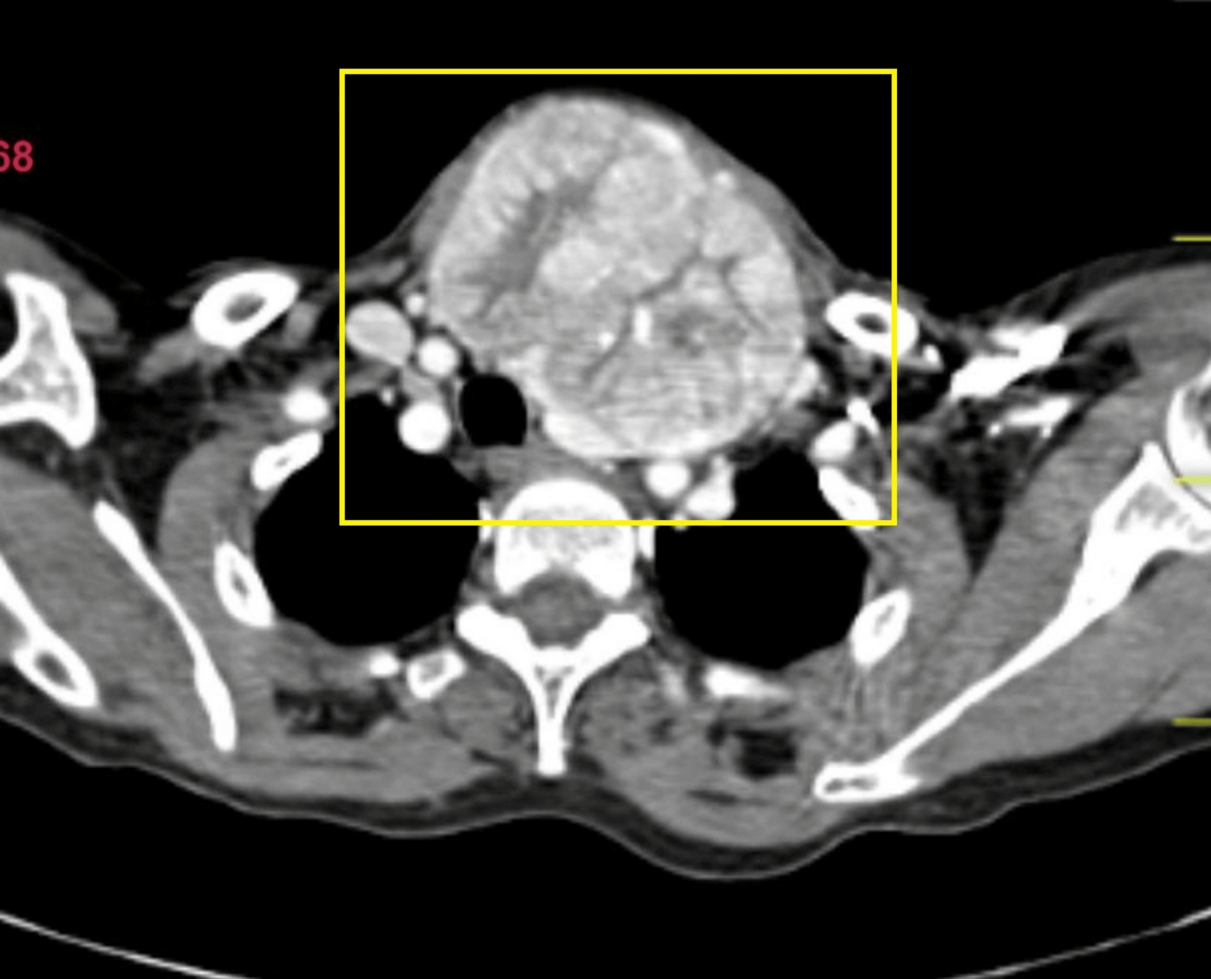
CECT showing thyroid nodule axial view showing heterogeneously enhanced soft tissue lesion with mass effect over trachea causing deviation of trachea towards the right side. CECT: contrast-enhanced computed tomography

**Figure 9:**
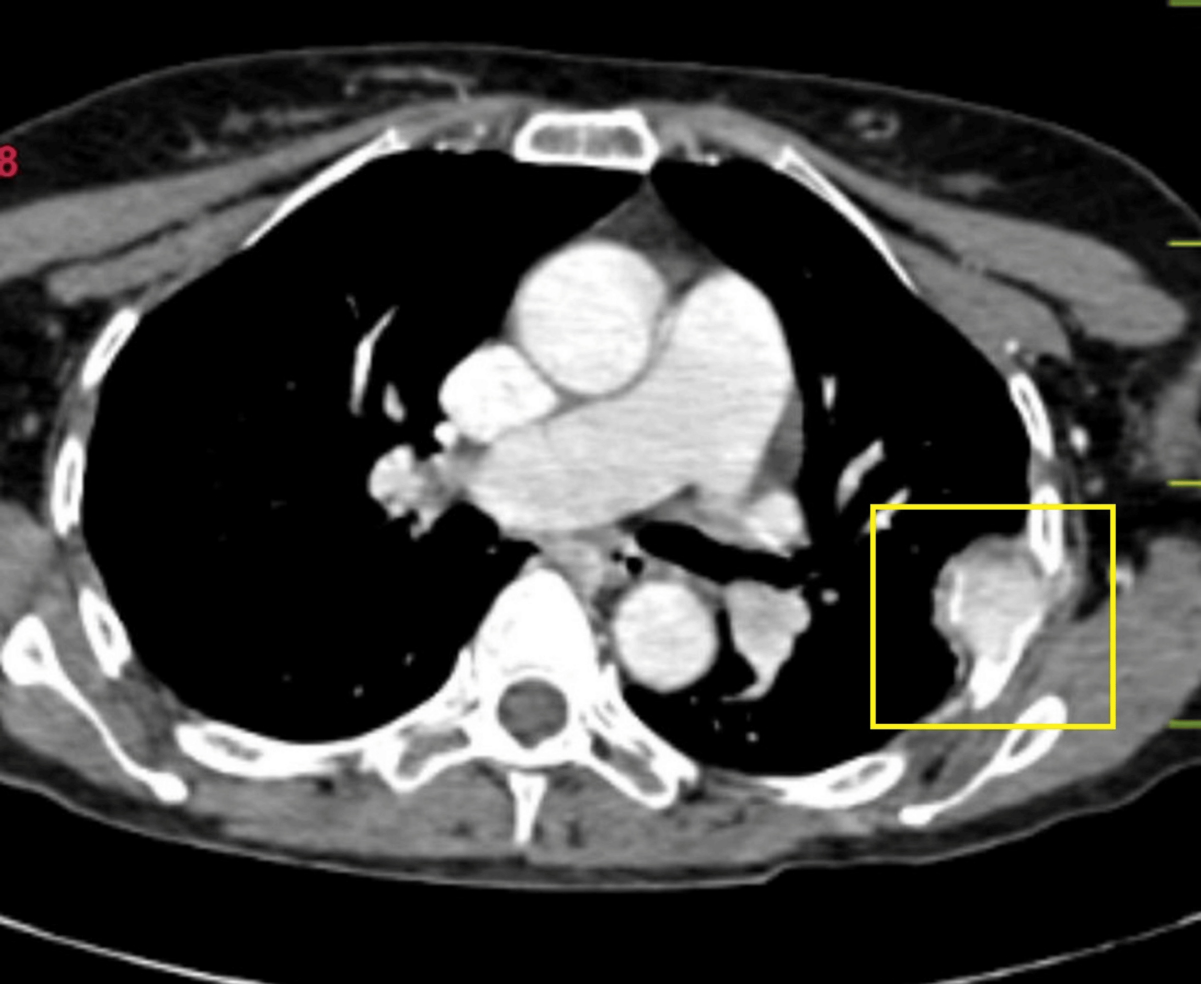
CECT showing the metastasis of the rib axial view showing enhanced soft tissue lesion arising from the fifth left rib with destruction of the involved rib CECT: contrast-enhanced computed tomography

**Figure 10:**
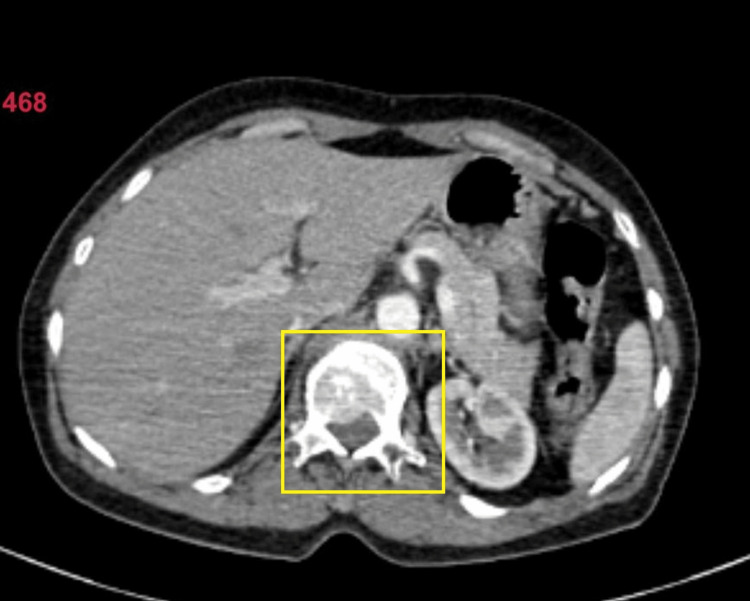
Computed tomography image showing lytic lesion of the vertebra (axial view)

Non-contrast CT scan was suggestive of an enlarged thyroid gland (Figure 5). Contrast-enhanced CT (CECT) of the neck and thorax revealed large heterogeneously enhancing soft tissue density arising from the left lobe of the thyroid, likely of neoplastic etiology. Heterogeneously enhancing soft tissue density from the fifth rib and lytic lesion in the body of the D12 vertebra were suggestive of metastasis (Figures 6-10).

**Figure 11:**
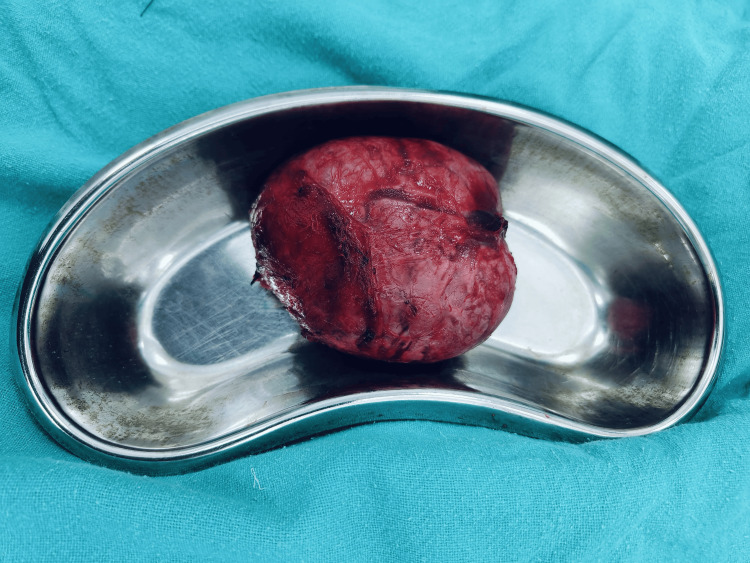
Excised specimen

**Figure 12:**
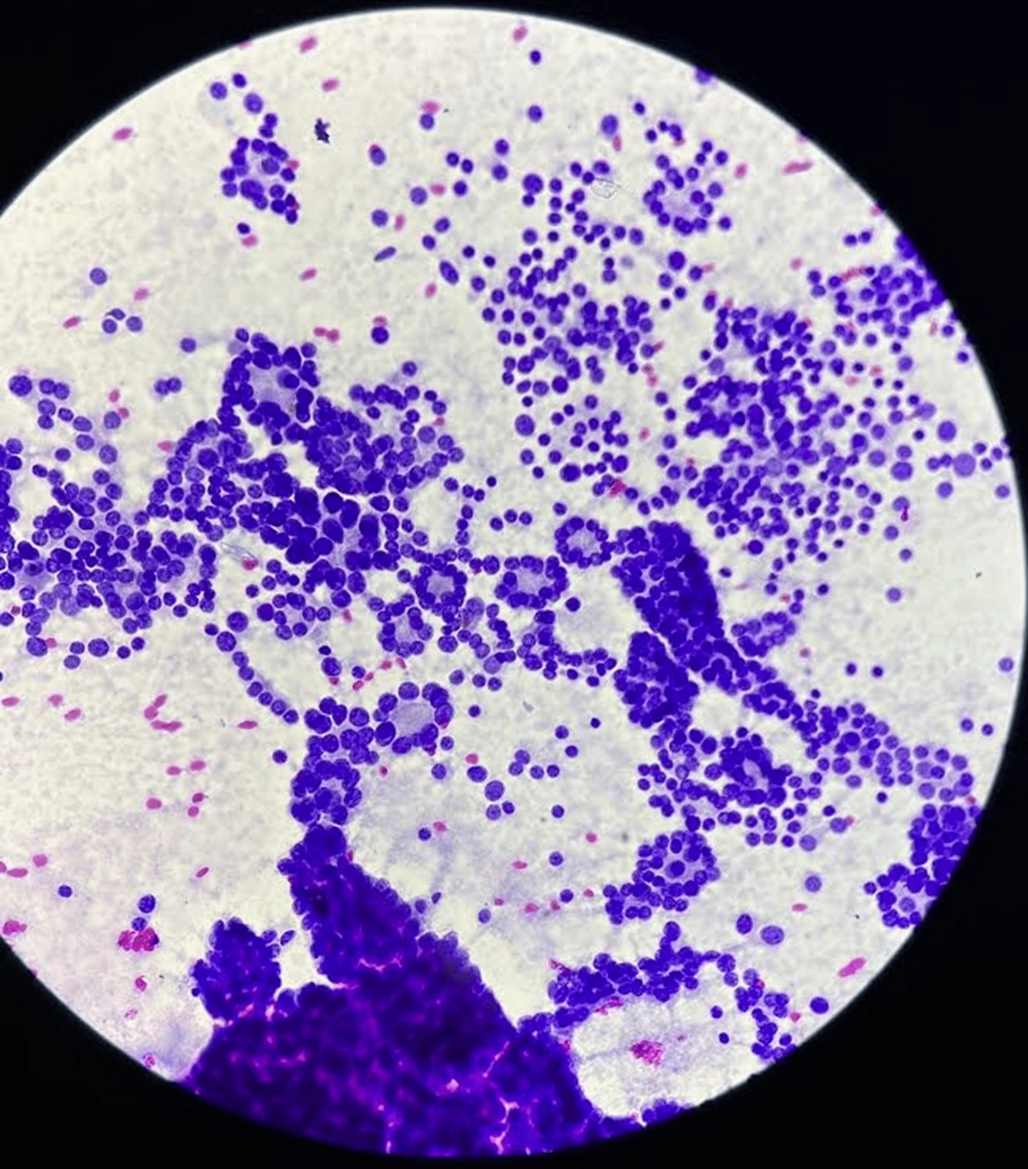
Hematoxylin and eosin staining of the histopathological slide of the excised specimen

On fine-needle aspiration cytology (FNAC) of the fifth rib, there was evidence of hyperchromic nuclei with mild pleomorphism, prominent micronuclei, and uneven chromatin, suggesting deposits of follicular thyroid carcinoma. The patient was subjected to a debulking surgery: total thyroidectomy with a modified radical neck dissection. The excised sample (Figure 11) was subjected to histopathological analysis which confirmed the diagnosis of follicular thyroid carcinoma (Figure 12). 

The patient was planned for palliative radiotherapy followed by chemotherapy. Postoperatively, tab levothyroxine (100 μg) was started. At the three-month follow-up, the patient had no fresh complaints, the scar site was noted to be healthy, and the metastatic workup showed no other significant metastasis. 

## Discussion

A neck lump may be a symptom of thyroid and parathyroid gland metabolic diseases, though there are a few inactive lesions too. Asymptomatic individuals might also develop a clinical presentation in the form of goiter. Thyroid cancer is the most frequent endocrine malignancy. Thyroid malignancy is common in females, constitutes nearly 3% of all cancers, and has been reported with a trend of increased incidence in the last few decades [2,4].

Follicular thyroid carcinoma, which makes up 15% of thyroid cancer cases, is more frequent in women over 50 years of age [3]. The current case presented with an enlarged 12 x 8 cm in the neck, which could be a result of neglected goiter over a period of time. Swelling of the neck might have underlying reasons such as lymphadenopathy and metastatic carcinomas [3,4]. Differential diagnoses can be concluded as papillary thyroid carcinoma, follicular adenoma, noninvasive follicular thyroid neoplasm with papillary-like nuclear features, poorly differentiated thyroid carcinoma, and metastatic non-thyroid carcinomas. 

FNAC continues to be a trustworthy investigation for head and neck swellings in the neck and the head. A study on neck swelling reported FNAC as an efficient and cost-effective tool with nearly no risk and minimum complications, leading to a conclusive diagnosis of the superficial mass [3,5,6]. The study reported the diagnostic accuracy of FNAC for these cancers, with a few cases as inconclusive. Overall diagnostic accuracy of the FNAC for thyroid cancers is reported as 85.14% [5]. Similarly, the FNAC report of this patient concluded follicular thyroid carcinoma and was further subject to its management.

Surgery is the most preferred treatment along with adjuvant therapy with radioactive iodine and follow-up to keep a check on the disease recurrence. Radioactive iodine (RAI) instead of chemotherapy is the treatment with debunking. RAI low doses for differentiated thyroid cancer have good survival rates [7,8]. For a patient with neck swelling/ thyroid swelling, the approach would be USG or CT scan followed by tissue diagnosis with the help of FNAC for confirmation of the diagnosis. Timely diagnosis and intervention are recommended in neck masses with a gradual increase in size, which might be helpful in the prevention of adverse outcomes.

## Conclusions

Any neck mass that is progressive in nature should not be ignored, as it might be the result of certain lymphadenopathy or malignancy, which might cause adverse outcomes if not timely addressed. Regular follow-up is important to monitor for any recurrence or spread. Overall, the prognosis of follicular carcinoma is generally favorable, with a good success rate of the treatment and long-term survival.
